# Vaccine-Associated Enhanced Disease and Pathogenic Human Coronaviruses

**DOI:** 10.3389/fimmu.2022.882972

**Published:** 2022-04-04

**Authors:** Cillian Gartlan, Tom Tipton, Francisco J. Salguero, Quentin Sattentau, Andrew Gorringe, Miles W. Carroll

**Affiliations:** ^1^ Wellcome Centre for Human Genetics and Pandemic Sciences Institute, Nuffield Department of Medicine, University of Oxford, Oxford, United Kingdom; ^2^ Research and Evaluation, UK Health Security Agency, Porton Down, Salisbury, United Kingdom; ^3^ The Sir William Dunn School of Pathology, University of Oxford, Oxford, United Kingdom

**Keywords:** vaccine-associated enhanced disease, COVID-19, SARS-CoV-2, coronavirus, vaccine, enhancement, safety

## Abstract

Vaccine-associated enhanced disease (VAED) is a difficult phenomenon to define and can be confused with vaccine failure. Using studies on respiratory syncytial virus (RSV) vaccination and dengue virus infection, we highlight known and theoretical mechanisms of VAED, including antibody-dependent enhancement (ADE), antibody-enhanced disease (AED) and Th2-mediated pathology. We also critically review the literature surrounding this phenomenon in pathogenic human coronaviruses, including MERS-CoV, SARS-CoV-1 and SARS-CoV-2. Poor quality histopathological data and a lack of consistency in defining severe pathology and VAED in preclinical studies of MERS-CoV and SARS-CoV-1 vaccines in particular make it difficult to interrogate potential cases of VAED. Fortuitously, there have been only few reports of mild VAED in SARS-CoV-2 vaccination in preclinical models and no observations in their clinical use. We describe the problem areas and discuss methods to improve the characterisation of VAED in the future.

## Introduction

**Figure 1:**
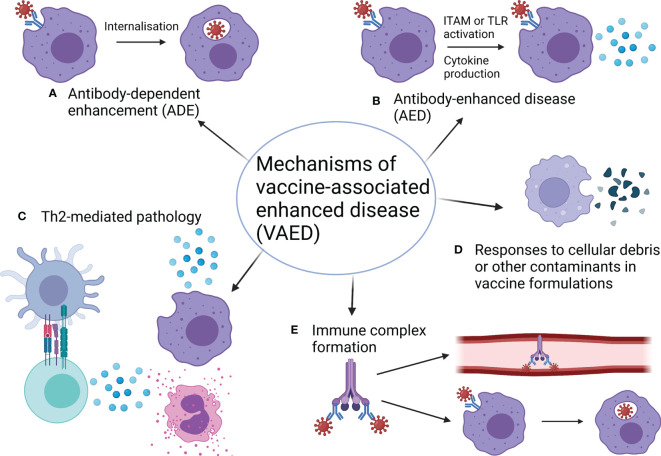
Mechanisms of vaccine-associated enhanced disease. **(A)** Antibody-dependent enhancement (ADE) occurs when antibodies increase the ability of a virus to infect cells (also see **Figure 2**). **(B)** Antibody-enhanced disease (AED) occurs when antibodies exacerbate inflammation, resulting in pathology (also see **Figure 2**). **(C)** Th2-skewed responses can be pathogenic for some infections and so vaccines that induce Th2 responses in this case can cause pathology. Usually Th2 pathology is associated with eosinophil infiltration. **(D)** Components of vaccine formulations such as bovine serum albumin (BSA) and cellular debris can mediate pathogenic cellular responses to these components when encountered again as contaminants in the challenge material. While these components are normally removed during vaccine preparation, some preclinical studies have not included appropriate washing and centrifugation steps to facilitate this. **(E)** Immune complexes between viral proteins, antibodies and/or complement can lead to a build-up of deposits in blood vessels and organs or facilitate enhanced uptake of virus through myeloid cells, causing ADE. Both of these outcomes can enhance pathology. Made with BioRender.com.

Vaccine-associated enhanced disease (VAED) is a rarely-observed phenomenon whereby vaccination promotes immune responses that exacerbate the disease caused by subsequent infection with the associated pathogen. VAED has been observed in humans in three vaccine trials, for vaccines against dengue virus, respiratory syncytial virus (RSV), and measles (1–3). Of these vaccines, only the dengue vaccine has been approved, under particular conditions to avoid inducing VAED (4). In this review, we outline the mechanisms of VAED (**Figure 1**), how it is characterised, critically evaluate the evidence surrounding VAED with a focus on pathogenic coronaviruses, and propose a framework which can be used to investigate VAED. While VAED is usually associated with antibody responses, there are also potential roles for T cells and complement, each of which will be discussed. To note; VAED is sometimes also referred to as vaccine-associated enhanced respiratory disease (VAERD). It can be difficult to distinguish between VAED and vaccine failure, however the Coalition for Epidemic Preparedness Innovations (CEPI) have recently developed a case definition for VAED (5). The guidelines put forward by this group of experts can be used to investigate potential cases of VAED in future clinical trials and may also help to inform such investigations in preclinical models.

**Figure 2:**
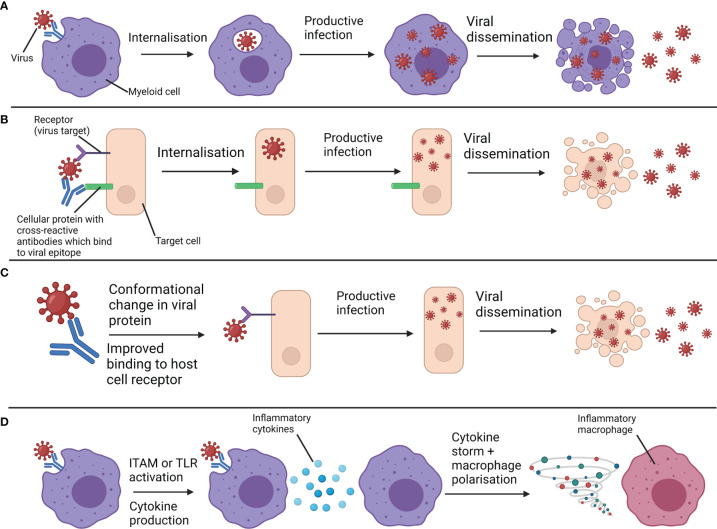
Overview of antibody-dependent enhancement (ADE) and antibody-enhanced disease (AED). **(A)** Non-neutralising antibodies or sub-neutralising antibody concentrations bind to viruses and interact with Fc receptors on myeloid cells. This facilitates the internalisation of viruses. Viruses that can productively infect myeloid cells can proliferate and spread following their uptake, enhancing infection. This is a form of ADE. **(B)** Cross-reactive antibodies bind to both virus and host cell components, bringing viruses in close contact with their receptor. Receptor-mediated uptake and enhanced infection follows. This is another form of ADE. **(C)** Antibodies against a particular epitope drive a conformational change in a viral protein which enhances infection through improved binding to the host cell receptor. This is another form of ADE. **(D)** Antibodies bound to virus interact with Fc receptors on myeloid cells and either activate immunoreceptor tyrosine-based activation motifs (ITAMs) associated with these receptors, or facilitate viral uptake and subsequent activation of endosomal toll-like receptors (TLRs). Through either of these mechanisms, inflammatory cytokines and chemokines are produced, exacerbating inflammation to a pathogenic extent and polarising myeloid cells towards more inflammatory phenotypes. These are forms of AED. Productive infection of myeloid cells is not required for this mechanism. Created with BioRender.com.

Antibody-dependent enhancement (ADE) is arguably the most well-understood mechanistic explanation of VAED, although in many cases it may not be the major contributor to VAED. ADE describes how antibodies enhance uptake of a virus into cells that can facilitate productive infection and viral dissemination (**Figure 2**). ADE has been best characterised in dengue virus reinfections and has been a major challenge in dengue virus vaccine development (6). ADE can be caused by binding of non-neutralising antibodies or sub-neutralising concentrations of antibodies. The former is an issue of antibody quality, while the latter is an issue of quantity. Both of these mechanisms result in virus uptake by phagocytic cells through Fc gamma receptors (FcγRs) on myeloid cells - including monocytes, macrophages and dendritic cells (DCs) - without neutralising the virus to protect against further infection. For viruses that can proliferate and propagate through infection of phagocytes, such as dengue virus, these mechanisms worsen disease outcomes. However, even in cases where viruses can mediate ADE, many antibodies provide a background of protection and only a minority of antibodies will contribute to ADE. Where antibody titre is the primary correlate, there is thought to be a range at which antibody concentrations are pathogenic rather than neutralising, as shown with dengue infection (7). At very low levels, there may not be enough antibody binding to facilitate ADE and at very high levels, antibodies may be able to neutralise viruses by preventing viral proteins binding to their receptor targets.

While ADE concerns enhancement of viral entry into susceptible cells, another term, antibody-enhanced disease (AED), concerns enhancement of immunopathology (see **Figure 2**). Sometimes, AED is also referred to as “ADE of disease” or “enhanced respiratory disease”. In this review we will use the terms “ADE” and “AED” separately. AED occurs when antibodies bound to a virus also bind to FcγRs of myeloid cells and increase inflammation to such an extent as to cause immunopathology. The increase in inflammatory cytokine production can be due to activation of immunoreceptor tyrosine-based activation motifs (ITAMs) through FcγRs (8) or activation of endosomal toll-like receptors (TLRs) by pathogen-associated molecular patterns (PAMPs) on a pathogen following antibody-mediated uptake. It is important to note that while FcγR binding or activation can be pathogenic in an ADE or AED context respectively, FcγRs also play a significant role in antiviral immunity, sometimes more than potent neutralising activity (9). The level of fucosylation of IgG1 antibodies is known to impact their ability to bind to and activate the activatory receptor FcγRIIIa, with lower fucosylation associated with stronger binding (10). Immune complexes can also contribute to ADE or AED. Immune complexes are formed by antibody-antigen aggregates and sometimes also involve binding of complement binding proteins. These complexes can be either protective or pathogenic, as they can stimulate antigen presentation and protective immune responses or be deposited in blood vessels and organs and trigger pathogenic inflammation (11). Immune complexes can also bind FcγRs to facilitate ADE (12).

At the onset of the COVID-19 pandemic there were significant safety concerns with the development of severe acute respiratory syndrome coronavirus 2 (SARS-CoV-2) vaccines (13), particularly given prior reports of VAED in preclinical models of SARS-CoV-1 and Middle East respiratory syndrome (MERS) vaccination. These studies will be a focus of this review. Animal models are essential for investigating VAED. However, progression of infection and severity of disease can vary widely between species and so different animal models have particular advantages in terms of immunological and pathological insights, although none completely mimic the human case (14–16). Beyond physiology, the molecular mechanisms that characterise coronavirus disease 2019 (COVID-19) progression can also vary between species. For instance, the gene encoding ACE2 is an interferon-stimulated gene (ISG) in human cells but not in murine tissue (17). This means that interferon induction in response to SARS-CoV-2 will likely have quite different pathological consequences in humans compared to mice. Fc receptor functions also vary widely between species, with humans exhibiting features absent in other species (18). This has implications for the study of the antibody-mediated elements of VAED in animal models. Overall, non-human primate (NHP) models are likely to provide the most relevant pathological insights to the human case, given similarities in physiology and immune mechanisms, although both rhesus and cynomolgus macaques both present only mild to moderate forms of COVID-19 (16). As many VAED concerns have been associated with formalin-inactivated vaccines, we will also discuss recent studies examining formalin-inactivated SARS-CoV-2 based vaccines in preclinical models, which were designed with the goal of stimulating VAED (19, 20). In order to investigate VAED in animal models, *in vivo* pathology studies are essential as disease progression and its consequences are difficult to determine from serological and other *in vitro* data alone.

## Background

Vaccines against dengue virus and RSV have demonstrated VAED in humans. While the mechanism of VAED is well-characterised as being mediated by ADE in dengue virus, the mechanisms behind VAED in RSV are less clear (**Table 1**).

**Table 1:** An overview of ADE, AED and VAED concerns relevant to dengue virus and RSV.

Virus	Summary of ADE, AED and VAED concerns	References
Dengue virus	ADE has been characterised both *in vitro* and *in vivo*. ADE through FcγRs on myeloid cells is thought to contribute to enhanced infection both *in vitro* and *in vivo*. ADE *in vivo* is mediated by poorly neutralising cross-reactive antibodies at particular concentrations Antibody responses to different serotypes of dengue virus generate cross-reactive antibodies that can be poorly neutralising, contributing to ADE. Another mechanism of ADE has been observed *in vitro*, where antibodies bind a cross-reactive epitope on host cells, although this mechanism has not been completely elucidated. Afucosylation of antibodies in dengue infection is also thought to contribute to ADE *in vivo* through higher affinity binding to FcγRs. Despite ADE, antibodies are also important in preventing dengue infection. VAED has been observed in seronegative individuals in response to a tetravalent dengue vaccine, but efficacy was observed in seropositive individuals.	(1, 6, 7, 21–25)
Respiratory syncytial virus (RSV)	VAED following formalin-inactivated RSV vaccine contributed to hospitalisation and two deaths in seronegative individuals. The mechanism of VAED following the inactivated RSV vaccine is debated, but is thought to be at least partially mediated by immune complex deposition in the lungs and pathogenic Th2 responses. The role of eosinophils with VAED in this vaccine has been debated and Th2 cells are more likely to contribute to pathology. Vaccinia virus expressing G protein has been used to replicate VAED in animal models, although the mechanism of VAED may differ from that seen in the formalin-inactivated RSV vaccine in humans. There are several potential mechanisms of Th2 polarisation with the formalin-inactivated RSV vaccine, including the use of alum as an adjuvant, carbonyl groups formed by formalin inactivation, formation of immune complexes with complement and virus by poorly neutralising antibodies. Recently, several vaccine platforms have demonstrated efficacy without VAED and several vaccine platforms are in phase III clinical trials.	(2, 26–36)

### Antibody-Dependent Enhancement

Dengue virus provides a clear example of VAED through ADE, enabled by its tropism for monocytes and macrophages in particular (6). As a consequence of ADE, secondary infections with a different serotype of dengue virus are much more pathogenic than primary infections (37). This is because many cross-reactive antibodies in the secondary infection lack neutralising activity but can still bind to the virus and assist its entry into FcγR-expressing phagocytes. Ramadhany et al. found that engineering of the Fc region on antibodies, which changes the antibody subclass, affects binding to FcγRs and subsequently the extent of ADE observed (38). The mutations that increased or decreased ADE activity sometimes depended on the cell type being infected, as different cell types express different levels of FcγR subtypes and other mutations decreased ADE in more than one cell type (38).

A monocyte-independent mechanism of ADE has also been proposed in dengue, although it remains to be seen if this occurs with other viruses. This mechanism requires molecular mimicry between virions and host cell components (self-antigens), such as the prM protein in dengue virions and heat shock protein 60 (HSP60) (21). However, the mechanism has not been completely elucidated and may involve conformational changes in virion proteins that mediate cell entry or simply bringing virions in close proximity with target cells so that they can interact with their receptor. As with other forms of ADE, its significance depends on the concentration of enhancing antibodies vs the concentration of neutralising antibodies. A bispecific antibody targeting host cells and dengue virus has provided evidence for this FcγR-independent form of ADE in dengue *in vitro* (22). Recent findings suggest that as antibody levels increase in dengue infection, the severity of ADE declines, as more neutralising antibodies are produced than non-neutralising antibodies (23). This suggests that only low levels of cross-reactive antibodies will cause ADE. While antibodies in dengue can contribute to ADE, antibodies are also important in preventing dengue replication, at least at the appropriate concentrations (24). An intermediate concentration range of cross-reactive antibodies is thought to contribute to ADE because higher titres contribute to neutralisation, while titres below this range are insufficient to cause ADE (7).

From dengue virus studies, it is apparent that several conditions can contribute to the development of ADE. Firstly, there are several serotypes of dengue circulating, each with antigenic variability. Secondly, binding of antibodies to these variable antigens can facilitate productive infection of myeloid cells through FcγRs Thirdly, subsequent infections with serotypes that differ from the first serotype encountered will provoke memory B cell responses which produce cross-reactive antibodies with non-neutralising or poorly-neutralising activity, facilitating productive infection of myeloid cells. Finally, when antibody levels wane after infection or vaccination, sub-neutralising concentrations of antibodies can enhance disease rather than prevent it (1, 7). Another factor that may contribute to ADE in dengue virus infection is the level of fucosylation of anti-dengue virus antibodies (25). Bournazos et al. found that dengue infection induced IgG1 afucosylation, which was associated with worsened disease outcomes upon secondary infection. Interestingly, the authors found no association between neutralising activity or antibody titres and severity of infection (25), contradicting the aforementioned idea that particular concentrations of non-neutralising antibodies are the primary contributors to disease severity. Afucosylation of IgG1 increases its binding affinity to FcγRIIIa (10). Therefore, afucosylated anti-dengue virus antibodies in secondary infection may improve the ability of the virus to invade monocytes through ADE. Bournazos et al. did not observe afucosylation of antibodies in patients infected with West Nile virus or Zika virus, which are other flaviviruses (25). While West Nile virus and Zika virus pathogenesis is exacerbated by monocytes, ADE is not thought to play a major role in the pathogenesis of either of these viruses (39–41).

Efficacy trials of a three-dose tetravalent dengue vaccine (CYD-TDV) demonstrated that serostatus upon vaccination determined whether the vaccine was protective or caused enhanced disease (1). Those who were seropositive for dengue prior to vaccination demonstrated lower levels of hospitalisation compared to controls, while those who were seronegative prior to vaccination demonstrated higher levels of hospitalisation when compared to controls. This phenomenon was not dependent on the serotype that vaccinated individuals were infected with, but was most prevalent in those infected by serotype 2. CYD-TDV administration was also associated with higher risks of severe thrombocytopenia (1). However, the vaccine has been shown to be safe and effective in seropositive individuals and has been approved for use in seropositive individuals aged 9 or above in many countries and the European Union (4).

ADE has also been observed following infection with the filoviruses Ebola virus (EBOV) and Marburg virus (MARV) *in vitro* and *in vivo* (42–45). In line with these findings, ADE has also been found to occur in humans regardless of serum antibody affinity, class or specificity and is instead dependent on antibody concentration, with sub-neutralising concentrations of antibodies enhancing infection (46). This study also found that antibodies with high affinity for FcγRs contribute more to ADE, although antibodies with low affinity for FcγRs can also make a contribution, consistent with the findings by Ramadhany et al. in relation to dengue virus (38). As with dengue, neutralising antibodies are important in protection against Ebola virus disease (EVD) (47), so in vaccination it is important to induce effective antibody titres to ensure protection and not disease enhancement. However, in rhesus macaques, it has been found that passive immunotherapy, involving transfer of convalescent plasma from EBOV-immune macaques to naïve macaques, can lead to enhanced infection, causing viral titres at death to rise over 100-fold above controls (48). Monoclonal antibodies have also been found to enhance viral replication in EBOV infection, unless used at high concentrations, when they become protective (46, 49–52). In humans, convalescent plasma was not associated with improved survival in treatment of Ebola infection (53).

### Antibody-Enhanced Disease and Th2 Pathology

RSV and measles are paramyxoviruses that have been linked with VAED and this has been reviewed elsewhere (26, 27, 54). Both viruses cause moderate symptoms in most infected people but are more likely to become severe or fatal in young children. In 1969, Kim et al. published a study on a formalin-inactivated alum-adjuvanted vaccine for RSV, which became the first study that demonstrated VAED in humans (2). Kim et al. reported that 80% of the vaccinated cohort that became infected required hospitalisation, compared to 5% of the infected control group and two young children in the vaccinated group died as a result (2). As with dengue, VAED was observed in previously seronegative individuals and not in those who were seropositive before vaccination (26). While much research has gone into what went wrong, there is still no licenced vaccine for RSV. Fears of VAED have delayed RSV vaccine development, so understanding the phenomenon is paramount to ensure rapid development of safe and effective vaccines (13). VAED in the inactivated RSV vaccine is thought to have been mediated by immune complex deposition in the lungs and pathogenic Th2 responses, which caused eosinophil infiltration of the lungs (26).

As well as the formalin-inactivated vaccine, vaccinia virus expressing the G protein (which RSV uses to attach to host cells) has been widely used to replicate the eosinophil infiltration seen in VAED pathology (26, 28, 29). While the goal of this research was to dissect a mechanism of VAED, it is unknown whether VAED in the vaccinia virus vaccines and the formalin-inactivated vaccine is mediated by the same mechanism, as pointed out by Acosta et al. (26). These authors have also pointed out that enhanced disease in cotton rat (55) and bovine (56) models does not involve eosinophils and that the role of eosinophils in mediating VAED has been questioned (30). Knudson et al. demonstrated using a formalin-inactivated alum-adjuvanted vaccine that it is Th2-biased CD4+ T cells that mediate VAED and not eosinophils or antibody levels (30). The role of CD4+ T cells and their production of IL-4 and IL-10 had also been highlighted many years earlier (57, 58), but only more recently could the eosinophilia that followed be ruled out as the cause of pathology (30). Consistent with the Th2 immune phenotype, the authors also noted a lack of CD8+ T cell induction by a formalin-inactivated RSV vaccine. Therefore, eosinophil infiltration may be linked to Th2 pathology in some cases without being the mediating factor of the enhanced disease. The role of eosinophils in VAED more generally may vary depending on the pathogen in question, the animal model and the characteristics of the vaccine preparation.

While immune complexes and weakly neutralising antibodies have also been suggested to contribute to VAED in RSV (31, 32), they do not have as convincing a mechanistic explanation as Th2-biased CD4+ T cells (30). As for what mediates the Th2 bias, there are several potential explanations (26). It is possible that the use of aluminium hydroxide (alum) as an adjuvant in many RSV vaccines could explain Th2 polarisation, as alum is a known Th2-skewing adjuvant (33).The Th2-skewing effect of carbonyl groups created by formalin inactivation itself could also cause the pathology seen in the formalin-inactivated RSV vaccine (34). Alternatively, a particular peptide in the G protein may cause the Th2 bias that leads to pathology and the associated eosinophilia (29). Formalin inactivation alone cannot explain Th2 polarisation, as studies using isolated G or F glycoproteins without formalin in BALB/c mice and cotton rats respectively also demonstrated this effect (59, 60). Both of these studies used formulations containing alum. Immune complexes could also possibly contribute to Th2 polarisation through ligation of FcγRs on macrophages (35). While one study examined weakly neutralising antibodies as potential inducers of Th2 responses, the TLR agonists used in this study may offer an alternative interpretation of the results (32). Addition of TLR agonists to a formalin-inactivated RSV vaccine led to greater affinity maturation and enabled the vaccine to induce protective instead of disease-enhancing responses, however the authors note that T cell polarisation resulting from the addition of the TLR agonists may be another explanation for prevention of VAED (32). Alternatively, a defined level of antibody neutralisation may have been able to overpower the pathogenic effect of Th2 responses. It is also possible that weak neutralisation activity only allows antibodies to carry out the more pathogenic side of their activity, forming immune complexes through complement activation, which have been shown to promote VAED upon subsequent RSV infection (31), possibly due to Th2-skewing (35). Affinity maturation induced by previous natural infection may explain why seropositive vaccine recipients did not show enhanced disease (2), as their antibodies likely had greater neutralising activity.

The balance of correlates of protection vs the correlates of enhanced disease in RSV vaccination are poorly defined (61). A study in the cotton rat model demonstrates that the use of a Th1-biasing adjuvant with the F protein is not enough, and that high doses of antigen are also required to provide protection against pathology (60). This highlights the significance of having both appropriate T cell bias and dosage to minimise pathology and induce protective responses, although these correlates of protection have been difficult to quantify. In this study, despite low virus titres and induction of neutralising antibody levels previously found to be protective, pathology was observed in the low-dose group that received a Th1-biasing adjuvant (60). More recently, live-attenuated and vector platforms administered intranasally have appeared to provide protection without any VAED in children (36) and several vaccine platforms targeting pre-fusion F protein are currently in phase III clinical trials. Viral vector platforms induce Th1-biased immune responses (62), which may explain the lack of VAED.

VAED has also been observed in children who received a formalin-inactivated measles vaccine (3) and is thought to have a similar mechanism to VAED in RSV (54). In measles, the symptomatic disease mediated by the vaccine was characterised as ‘atypical measles’ and as in RSV, could be abrogated by neutralising antibodies induced by infection with live virus (54). This is the earliest known case of VAED in humans, although the RSV case was the first to be correctly characterised as VAED. Immune complex formation by non-neutralising antibodies and the subsequent deposition of these complexes are also thought to contribute to VAED in atypical measles (63).

If a virus cannot infect and replicate within myeloid cells (through ADE), AED can still occur. For AED, the infection of myeloid cells is replaced with induction of other antibody-mediated activities that exacerbate immunopathology. In the context of vaccination, antigenic variability in some cases of ADE/AED, such as that seen in dengue, could be replaced by the use of an antigen in the vaccine preparation that generates non-neutralising or sub-neutralising antibody responses. This could arise through use of an unintentionally modified and/or conformationally incorrect antigen. For instance, formalin inactivation can alter the structure of antigens through addition of cross-links, or the presence of reactive carbonyls may alter their Th bias, as discussed previously in the context of RSV (34). Alternatively, the use of inappropriate cell lines to produce vaccine antigens can lead to weakly neutralising antibody responses due to differences in post-translational modifications when compared to the case of human infection, which has been shown in influenza vaccination (64). While this has not resulted in VAED, this mechanism could theoretically cause VAED in other viral infections.

## Vaccine-Associated Enhanced Disease in Pathogenic Coronaviruses

ADE in coronaviruses infections has been best-characterised in feline infectious peritonitis (FIP) (65), which affects cats and not humans. Like dengue virus, FIP virus (FIPV) productively infects macrophages (66). Unlike dengue virus, re-infection with the same serotype of FIPV can lead to ADE (67), possibly caused by sub-neutralising antibody concentrations. However, unlike dengue virus, the more severe pathogenesis of FIP arises when the spike protein of FIPV mutates within the host to allow infection of macrophages (68). While some FIPV vaccine efforts have demonstrated efficacy, others have caused VAED (69). In human coronaviruses, there is conflicting evidence concerning ADE and AED in particular (**Table 2**) (100). We will review the evidence for and against, focusing on potential mechanisms and *in vivo* pathology. Some authors have made claims of ADE in human coronavirus infections after providing evidence for their antibody-mediated entry of myeloid cells. However, without productive infection within these cells, it is unlikely that any pathological consequences *in vivo* caused by ADE can occur. Antibody-mediated entry of viruses into myeloid cells is therefore not sufficient evidence for ADE, as viruses are often eliminated by macrophages through this mechanism, known as antibody-dependent cellular phagocytosis (ADCP). In order to avoid AED, vaccines must generate responses against appropriate targets which provide effective neutralisation, such as the viral protein used to interact with cellular receptors. In coronaviruses, the receptor-binding domain (RBD) of the spike is the most appropriate target, as blocking it prevents binding to its cellular target. Some of the VAED in preclinical vaccines against coronaviruses may have resulted from responses generated against other structural proteins or modified spike protein such that the antibodies generated against it are sub-neutralising. Alternatively, VAED may sometimes be explained through inappropriate skewing of immune responses towards inflammatory phenotypes that exacerbate pathology or presence of contaminants in vaccine preparations.

**Table 2:** An overview of ADE, AED and VAED concerns in pathogenic coronaviruses.

Virus	Summary of ADE, AED and VAED concerns	References
Feline infectious peritonitis virus (FIPV)	The spike protein undergoes an infection-enhancing mutation within the host to infect macrophages. ADE is observed, even upon re-infection with the same serotype. Possible VAED has been noted and there is currently no effective vaccine.	(65–69)
Middle East respiratory syndrome coronavirus (MERS-CoV)	ADE has been observed at particular antibody concentrations. However, only low levels of productive infection of myeloid cells have been reported. AED has been observed *in vitro* through cytokine induction and *in vivo* in rabbits upon reinfection, possibly owing to complement activation and a lack of neutralising antibodies, which are protective. Possible Th2 pathology has been observed in mice that received a gamma radiation-inactivated vaccine or a UV-inactivated vaccine. In contrast, a viral vector vaccine in mice has been shown to be protective. Successful vaccination of macaques has been demonstrated without observations of enhanced pathology.	(70–76)
Severe acute respiratory syndrome coronavirus 1 (SARS-CoV-1)	While antibodies can enhance viral entry, they lead to abortive infections of myeloid cells. Anti-spike antibodies have been shown to provoke inflammatory cytokine production and macrophage skewing towards inflammatory phenotypes *in vitro*. Formalin-inactivated vaccines are protective but may also induce pathology. TLR agonists may be able to protect against pathology induced by inactivated vaccines. Antibody responses mounted against a particular epitope (S_597-603_) might induce AED in macaques. Antibody responses against the RBD are thought to be protective, while responses against other components of the spike, such as the nucleocapsid, may cause pathology.	(77–93)
Severe acute respiratory syndrome coronavirus 2 (SARS-CoV-2)	Antibodies against particular epitopes can enhance infection *in vitro via* a novel ADE mechanism in ACE2-transfected HEK293T cells, which lack TMPRSS2. Antibodies that enhance infection *in vitro* do not enhance infection *in vivo*. No strong evidence of ADE, AED or VAED following at preclinical or clinical vaccine evaluation. In studies designed to induce VAED in ferrets, Syrian hamsters and macaques, protection against disease was observed and only transient pathology was noted in ferrets.	(19, 20, 98, 99)

Preclinical vaccine studies with other coronaviruses raised concerns for the development of SARS-CoV-2 vaccines, owing to reports of vaccine-enhanced disease (VAED). While Feline infectious peritonitis virus, MERS-CoV and SARS-CoV-1 have shown evidence for ADE, AED and/or VAED in animal models, this has never been demonstrated in humans. SARS-CoV-2 preclinical studies did not highlight VAED as a concern.

In MERS, the mechanism of antibody-mediated entry into myeloid cells is thought to involve a conformational change in the spike protein upon antibody binding to the RBD, but not to other sites (70). This uptake is mediated by the same pathway as that induced by its cellular target, DPP4. Similar to the *in vivo* ADE dengue virus findings mentioned previously (7), it was found that the extent of viral entry was dependent on intermediate concentrations of antibodies against MERS-CoV *in vitro* (70). However, as only low levels of productive infection of macrophages and dendritic cells have been reported (71, 72), anti-MERS-CoV antibody concentrations are not linked to pathogenesis to the same extent as anti-dengue virus antibody concentrations (7). When anti-MERS-CoV antibodies are seen to enhance pathology, it is through AED. MERS-CoV has been found to induce inflammatory cytokines and chemokines in infected macrophages (71), which contribute to the cytokine storm associated with the immunopathology of severe MERS-CoV infection (101). MERS-CoV reinfection in rabbits has been shown to cause severe immunopathology, partly owing to the lack of neutralising antibodies, which were only produced following this second exposure, as well as the disease-enhancing activity of non-neutralising antibodies (73). The authors suggested AED *via* complement activation rather than ADE, as no increase in viral load was seen during the reinfection, demonstrating *in vivo* that the extent of productive infection of macrophages in MERS is not involved in pathogenesis. Importantly, the authors found neutralising antibodies to be protective (73), thus highlighting the importance of including appropriate antigens in vaccines to induce neutralising rather than non-neutralising antibodies which may be pathogenic.

There have also been suggestions that immunisation against MERS can cause Th2 immunopathology in mice (74). The authors used transgenic mice with the human MERS virus receptor DPP4. Immunohistochemical (IHC) staining was used to visualise eosinophils but published images showed very high background, and as the authors did not show haematoxylin and eosin (H+E) stained images, it is difficult to interpret whether the MBP-positive cells are eosinophils as they are claimed to be. Immunisation of mice with a parainfluenza viral vector was found to be effective and did not cause pathology, while a UV-inactivated MERS-CoV vaccine was protective but also associated with immunopathology (75).

Successful MERS vaccination has been observed in rhesus macaques with the use of the ChAdOx viral vector platform (76), which is also used in a widely-administered and effective human SARS-CoV-2 vaccine (102). Rather than enhancing disease, as might be expected from the aforementioned *in vitro* studies and *in vivo* murine studies, this MERS vaccine protected the macaques from respiratory pathology across six different MERS-CoV strains (76). This demonstrates the importance of carrying out *in vivo* histopathological analysis in appropriate models when studying VAED. This vaccine has been assessed in phase 1 trials with promising safety and immunogenicity data (103) but any concerns of VAED can only be monitored if larger trials are conducted where vaccinees are exposed to MERS-CoV infection. Safe and effective vaccination against MERS virus using a modified vaccinia Ankara (MVA) viral vector has also been observed in mice (104, 105) and in dromedary camels, the reservoir host of the virus (106). The MVA vector has also been assessed in phase 1 trials (107).

With regards to SARS-CoV-1, a closer relative of SARS-CoV-2, antibodies have been reported to enhance *in vitro* infection of myeloid cells (77, 78) but crucially these cases were non-productive or ‘abortive’ infections, meaning the viral particles were unable to replicate within these infected cells and disseminate further. Instead of antibody-macrophage interactions enhancing disease, macrophages in SARS-CoV-1 infection appear to be essential for antibody-mediated viral clearance, as demonstrated in mouse models (79). In this study, the importance of ADCP by macrophages was highlighted by the fact that neutralising activity was not essential for viral clearance and species-matched Fc receptors were required for clearance (79). However, in SARS-CoV-1 vaccination, the story is more complex.

Liu et al. demonstrated that vaccination of Chinese macaques with an MVA vector encoding the SARS-CoV-1 spike glycoprotein induced high levels of neutralising antibodies that reduced viral loads following challenge. However, histopathological examination revealed that these animals had greater lung damage following challenge compared to controls (80). Through adoptive transfer of vaccine-induced neutralising antibodies to unvaccinated macaques, Liu et al. showed that anti-SARS-CoV-1 spike antibodies conferred more severe lung damage compared to controls upon challenge. The features of diffuse alveolar damage (DAD) described include the presence hyaline membranes in Figure 1. However, it is not clear if hyaline membranes are present in the images in this figure and the pathology may be incorrectly interpreted. Similarly, in Figure 2, the pathology that the authors allude to in the figure legend is difficult to observe and the scoring system used is unclear. The enhanced pathology correlated with infiltration of inflammatory macrophages and rising serum IL-8 levels is much clearer, as areas of interest are magnified (80). The presence of anti-spike antibodies skewed lung macrophages towards inflammatory phenotypes and away from wound-healing phenotypes found in higher numbers in controls (80). Consistent with these findings, sera from deceased SARS-CoV-1 patients demonstrated higher levels of neutralising antibodies and of inflammatory macrophages when compared to SARS-CoV-1 survivors and the antibodies from deceased patients could skew macrophages towards inflammatory phenotypes. The skewing effect was partially attributable to engagement of FcγRs (80). However, these skewing effects were noted in isolated monocytes from the infected macaques, and so this *in vitro* analysis lacks the context of other factors *in vivo* that may have contributed to AED in this case, such as formation of immune complexes following complement deposition.

In another study testing candidate MVA vector vaccines expressing SARS-CoV-1 spike protein or nucleocapsid, carried out in ferrets, neither vaccine was protective and the MVA-spike vaccine was reported to be associated with hepatitis (81). Less severe pathology was noted in the MVA-nucleocapsid vaccine and the MVA-spike vaccine that was associated with hepatitis also induced neutralising antibodies (81). However, for findings in ferrets it must be noted that hepatitis is often a background finding in experimental settings for this animal model, possibly caused by prior chronic viral infection with hepatitis E (82).

A live-attenuated mucosal vaccine against SARS-CoV-1, based on an attenuated parainfluenza virus vector expressing the spike protein, has shown efficacy in preventing viral shedding following SARS-CoV-1 challenge in African green monkeys (83). The vaccine was administered *via* both a nasal and intratracheal inoculation and no evidence of VAED was observed, although the study did not closely examine pathology. Another intranasal viral vector SARS-CoV-1 vaccine, using a recombinant adeno-associated virus platform, has also been shown to be protective in mice and was associated with less pulmonary pathology (84).

Formalin-inactivated SARS-CoV-1 vaccines have also demonstrated both protection (85) and pathology (86) in vaccinated macaques following challenge. Alongside possible VAED from a formalin-inactivated vaccine, Wang et al. found that human antibodies against a particular epitope, S_597-603_, on the spike protein could enhance infection of Vero E6 cells (derived from kidney cells extracted from African green monkeys), which lack FcγRs (86). Combining these findings with the pathology they observed in macaques, Wang et al. suggested possible ADE owing to the previously described mechanism of dual-specific antibody binding due to molecular mimicry between virion and host cell components (21) (**Figure 2B**), implying that the host cell component in question is present both in macaques and Vero E6 cells. While this phenomenon has been observed with dengue virus, it could also apply to SARS-CoV-1 and other coronaviruses. When a peptide (S_597-625_) containing this epitope was used to vaccinate macaques, it was found to cause more severe lung histopathology compared to other peptides and it was also not protective (86). A monoclonal antibody against this epitope, at a dose of 1.8 mg/kg, caused some pathology in the lungs following challenge, as well as macrophage infiltration and higher numbers of cells infected with SARS-CoV-1 (86). Antibodies against this epitope are likely non-neutralising because this region is not within the RBD. However, in this paper, the gross pathology is difficult to interpret, given the poor quality of the images showing abundant *post-mortem* artefacts. The H&E staining is also difficult to interpret as the authors focus on small areas with high magnification. Ideally, Wang et al. would have used virus detection and a robust histopathology scoring system or digital image analysis to interpret their findings. The study lacks an objective quantitative analysis for pathology observed by IHC, which would be useful and less prone to bias. A SARS-CoV-1 reinfection study of macaques could determine whether vaccine-induced antibodies are to blame for enhanced pathology following challenge or if neutralising antibodies resulting from natural infection also cause pathology in the same way. This could lay to rest whether the mechanism of enhanced pathologies seen in SARS-CoV-1 preclinical studies is due to poor quality vaccine preparations/design or whether it is the result of a viral AED mechanism. Further studies are required to determine the balance between neutralising and non-neutralising (or potentially enhancing) antibodies in re-infected vs vaccinated challenge models and how these antibody ratios relate to observed pathology.

While mouse models are not ideal for studying the pathology of coronaviruses, some murine studies have demonstrated signs of protection or enhanced pathology with SARS-CoV-1 vaccines. A SARS-CoV-1 vaccine that was double-inactivated using both formalin and ultraviolet (UV) light inactivation demonstrated signs of enhanced pathology associated with eosinophilic infiltration and provided poor protection against heterologous challenge in aged mice (87). Heterologous challenge involving infection with a lethal zoonotic virus (icHC/SZ/61/03-S) led to worsened pathology in young vaccinated mice compared to young unvaccinated mice. While immune infiltrates were noted, the observed pathology did not correlate with weight loss or mortality by day 4 post challenge. The authors hypothesise that anti-nucleocapsid responses contribute to the observed pathology. An alum-adjuvanted version of this vaccine protected young mice from both homologous and heterologous challenge but demonstrated eosinophil-associated pathology following heterologous challenge (87). The eosinophil-associated pathology in response to a UV-inactivated vaccine in mice can be overcome through the use of TLR agonists, which also maintain the protection observed in this vaccine (88).

Another study found pulmonary eosinophilic infiltrates in a variety of vaccines in Balb/c mice, however these mice were also ultimately protected against challenge (89). Tseng et al. demonstrated that Th2 immunopathology is induced by a variety of vaccines and hypothesised that non-neutralising antibody responses against nucleocapsid protein might explain much of the observed immunopathology, as shown previously (90, 91). This is because although the recombinant spike protein vaccine (SV) induced immunopathology, it was to a lesser extent than that observed in the other vaccines evaluated (89). Tseng et al. also point to a paper examining a spike-expressing vector which did not show immunopathology in mice and a nucleocapsid-expressing vector that did (91). The other vaccines included evaluated by Tseng et al. included a virus-like particle (VLP) vaccine, a double- inactivated vaccine (DIV), which was inactivated with both formalin and UV radiation and a whole virus vaccine inactivated with propiolactone and formulated with alum (BPV). The histopathology and IHC staining demonstrate eosinophilic infiltrates and pulmonary pathology in Balb/c mice given the SV, DIV or BPV vaccines two days following challenge. However, Tseng et al. do not quantify the pathology. Furthermore, the authors have not included an image of a bronchus, bronchiole and a large blood vessel for the H+E stain control, making it difficult to compare the histopathology between the control and challenged mice. In contrast to the study by Tseng et al. in which a spike protein vaccine demonstrated signs of immunopathology (albeit less than the other vaccines evaluated) (89), another subunit vaccine based on the RBD of the spike has demonstrated protection and no vaccine-associated pathology following challenge in mice (92).

A potential reason for observed ADE and AED in formalin-inactivated vaccines is that formaldehyde treatment may alter the structure of the spike protein through cross-linking such that the antibodies generated have reduced neutralising activity against wild-type SARS-CoV-1. The potential for this mechanism was demonstrated with a formalin-inactivated virus studied by our group, which then generated suboptimal vaccine responses, which we hypothesised was due to reduced exposure of the spike RBD following formaldehyde treatment (19). While this has not yet been shown to be true of the SARS-CoV-1 spike, a similar mechanism may be at play given the homology between the viruses. The impact of formalin inactivation will be described further in the SARS-CoV-2 section. Other forms of neutralisation may not alter protein conformations to the same extent. For instance, a SARS-CoV-1 vaccine containing beta-propiolactone-inactivated SARS-CoV-1 has demonstrated no AED-associated pathology in rhesus macaques, despite low levels of neutralising antibodies (93).

VAED in formalin-inactivated vaccines may also be caused by contamination of the vaccine preparation, with culture medium or cellular debris for example. Shaw and colleagues found that a formalin-inactivated RSV vaccine caused respiratory disease in the cotton rat model, which is widely used for investigation of RSV vaccine VAED following challenge, even when the RSV components were removed from the vaccine (94). Thus, it was found that the pathology was mediated by T cell responses to non-viral antigens, which included vaccine contaminants like bovine serum albumin (BSA). We hypothesised that we did not observe enhanced pathology in our NHP VAED study of a formalin-inactivated SARS-CoV-2 because of successful removal of such contaminants (19).

As previously mentioned, animal models have their obvious limitations and so the most convincing evidence for a lack of VAED in coronavirus vaccination would come from SARS-CoV-1 and MERS-CoV vaccine efficacy clinical trials. Phase 1 clinical trials found these vaccines to be safe and immunogenic and there were no reports of enhanced disease in vaccinated individuals (95–97), although vaccine effectiveness could only be assessed in phase III efficacy trials, which did not go ahead due to low infection rates as the epidemics were controlled. In an analysis of FcγRIIa polymorphisms on SARS-CoV-1 infection outcome, it was found that patients with a high-affinity FcγRIIa allele (H131) were more likely to survive and had reduced severity of disease (108). The low-affinity allele (R131) on the other hand was prevalent in higher proportions in patients who were hospitalised or who had died following infection. If AED contributed to pathology in natural infection with SARS-CoV-1, one would expect the opposite findings because in AED, engagement of activatory Fc receptors, such as FcγRIIa, is pathogenic. The findings instead indicate that FcγRIIa in SARS-CoV-1 may instead aid viral clearance through ADCP.

## Vaccine-Associated Enhanced Disease in the Context of SARS-CoV-2

Considering the aforementioned suggestions of AED in other coronaviruses discussed above, there has been much discussion around the potential dangers of AED in SARS-CoV-2 (109–112). As of yet there is no evidence of FcγR-mediated ADE in SARS-CoV-2 infection and unlike MERS, which demonstrated some evidence of productive infection of macrophages, SARS-CoV-2 does not productively infect macrophages (113, 114). While reinfection data in animal models and humans is lacking for other coronaviruses, infection of macaques with SARS-CoV-2 has been found to reduce disease severity upon reinfection, with reduced viral loads and high neutralising antibody titres noted (115, 116). There have also been no indications that the severity of reinfections with different variants can be exacerbated by antibody responses, as occurs with infections with different serotypes of dengue virus. However, a different mechanism of ADE has been proposed for SARS-CoV-2, involving antibodies against particular epitopes in the N-terminal domain (NTD) of the S1 component of the spike protein (98). These ‘enhancing antibodies’ have been shown to open the RBD, increasing its affinity for ACE2 and thereby enhancing infectivity. Each individual enhancing antibody is thought to bind to a particular site of the NTD on two different spike proteins at once to facilitate the opening of the RBD. Interestingly, these ‘enhancing antibodies’ have been found in both uninfected and infected individuals, with severe COVID-19 patients having a higher enhancing:neutralising antibody ratio (98). The authors also found that neutralising anti-RBD monoclonal antibodies could reduce the fold-change in ACE-2 binding conferred by enhancing antibodies, particularly at higher concentrations. However, this enhancing activity was not completely eliminated, up to a neutralising antibody concentration of 1ug/ml. It is important to note that this work was carried out *in vitro* and specifically in an ACE2-transfected HEK293T cell line (98), which lacks the SARS-CoV-2 co-receptor TMPRSS2, so does not mimic the prominent viral entry mechanism seen in natural infection (117). A recent study that used *in vitro* enhancing antibodies *in vivo* in mice and macaques found no evidence of enhancement of disease progression and instead found these antibodies to be protective across dose levels (99). The study found three of the 46 monkeys infused with enhancing bodies to have higher inflammation scores than controls and one monkey which had higher inflammatory cytokine levels in the bronchoalveolar lavage. This indicates that while enhancing antibodies may enhance infection *in vitro*, they may also sometimes cause an increase in lung inflammation, perhaps through an FcγR- and ITAM-mediated AED mechanism. In a vaccine context, these enhancing antibodies are not likely to be produced in large enough quantities to overcome the protection conferred by neutralising antibodies and T cells, which is consistent with the protection rather than enhancement observed in vaccine studies. Some other potential concerns of AED in COVID-19 come from studies of antibody fucosylation. The spike-specific and the RBD-specific antibodies of COVID-19 patients with more severe disease have been found to have lower fucosylation of their Fc domains (118), which is associated with increased binding to the activatory Fc receptor FcγRIIIa, hypothesised to cause immunopathology. However, *in vitro*, antibodies from COVID-19 patient convalescent plasma have been shown not to contribute to aberrant cytokine production by macrophages upon binding to FcγRIIa or FcγRIIIa (119). There is also no convincing evidence of AED contributing to pathology *in vivo* in SARS-CoV-2 vaccine studies, in contrast to the conflicting findings and misinterpretations of VAED in preclinical vaccine studies for SARS-CoV-1 and MERS-CoV vaccines.

**Figure 3:**
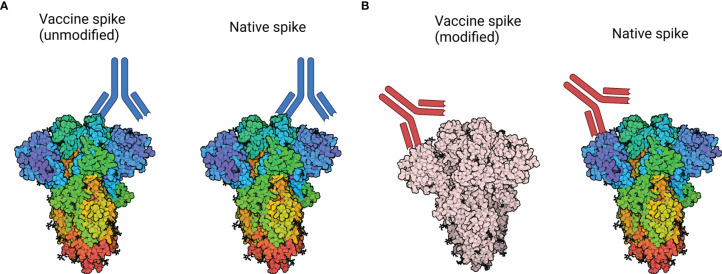
How modification of the spike protein could impact neutralising activity. **(A)** Native spike: Antibodies are generated against a neutralising epitope on the vaccine-derived spike protein, which closely represents the neutralising epitope found on circulating virus. As a result, neutralising activity against the vaccine-derived spike corresponds to neutralisation of circulating virus. **(B)** Modified spike: Antibody responses may be generated against a modified epitope, which no longer represents a neutralising epitope on circulating virus. As a result, antibodies produced in response to native spike may be sub-neutralising or non-neutralising and may contribute to VAED. Spike protein structure accessed through Protein Data Bank, PBD entry 6VXX (120). Created with BioRender.com.

As previously mentioned, we have demonstrated that formaldehyde treatment of the SARS-CoV-2 spike leads to cross-linking in this protein (19). We proposed that formaldehyde fixation secured the conformation of trimers in the spike protein such that approximately half of the trimers were in a ‘RBD down’ conformation, while the other half of the trimers were in the ‘RBD up’ conformation. The ‘RBD up’ conformation is also known as the prefusion conformation. By fixing the conformation with formaldehyde, the trimers are no longer free to sample both conformations, meaning the neutralising epitopes of the RBD will likely have lower immunogenicity due to their exposure being reduced in half of the trimers (19). As a consequence, antibody titres developed against SARS-CoV-2 in a formaldehyde-fixed vaccine may be sub-neutralising when challenged with live virus. While this mechanism can lead to AED in theory (**Figure 3**), we did not observe signs of VAED in rhesus macaques and only transient signs of pathology in ferrets, despite using a formaldehyde-inactivated vaccine formulated with Alhydrogel, designed to elicit VAED (19). Similarly, a study using a formaldehyde-inactivated vaccine in a Syrian hamster model found protective effects and no enhanced pathology after viral challenge (20). This is despite the use of a regimen designed to enhance disease and the Syrian hamster is a more severe disease model than the ferret or macaque (14, 15). Some protective effects were noted even if the vaccine was administered just before or shortly after infection, when antibody levels are more likely to be found at sub-neutralising concentrations and are less likely to have high affinities conferred by affinity maturation (20).

The spike protein epitope responsible for observed AED in a formaldehyde-inactivated SARS-CoV-1 preclinical vaccine study, mentioned previously (86), is also present in SARS-CoV-2. The amino acid sequence is LYQDVNC and is found at S_597-603_ in the SARS-CoV-1 spike protein and S_611-617_ in the SARS-CoV-2 spike protein. While formaldehyde inactivation may explain VAED to an extent, the enhancing effect of high doses of a monoclonal antibody against this peptide in SARS-CoV-1 (86) warrants investigation for similar phenomena in SARS-CoV-2. However, VAED has not been commonly observed in animal models and there are no reports in human trials of SARS-CoV-2 vaccines to date. While subunit vaccines containing the spike protein are immunogenic, subunit vaccines that use only the RBD of the spike are thought to require adjuvants in order to stimulate protective immunity (121, 122).

Beta-propiolactone inactivation of SARS-CoV-2 has been reported to cause viral aggregation at high concentrations, which can lead to a loss of antigenic potential, owing either to this aggregation or chemical modification of viral amino acids (123). However, the concentration (1:1000) at which this loss of antigenic potential was seen, was found to be much greater than the concentration of beta-propiolactone that is sufficient for inactivation. Beta-propiolactone-inactivated SARS-CoV-2 vaccines induce protective neutralising antibody responses without AED in macaques (124). Another preclinical study in macaques found such a vaccine to be safe and effective, with no AED despite low neutralising antibody titres in low-dose groups (125). A vaccine based on beta-propiolactone inactivation has been shown to be safe and effective (126) and is now in widespread use.

While an MVA vaccine for SARS-CoV-1 demonstrated possible AED in macaques (80), an MVA vaccine for SARS-CoV-2 protected against immunopathology following challenge and induces potent antibody and CD8 T cell responses in macaques (127). The MVA vaccine used in the latter study, MVA/S, expressed a membrane-anchored full-length spike protein and contained two mutations that ensure the spike is always in a prefusion (RBD up) conformation, meaning the RBD would always be completely exposed. This likely contributed to its potent immunogenicity. It is possible that this MVA/S vaccine approach generated antibodies with stronger neutralising capabilities and this could partially explain why VAED was observed in the SARS-CoV-1 MVA vaccine and not in the SARS-CoV-2 vaccine.

A DNA vaccine for SARS-CoV-2 has been shown to induce protective immune responses in rhesus macaques (128). Pathological analysis following challenge demonstrated protection in the majority of the animals in the one- and two-dose groups. However, one of the animals in the one-dose group had a much higher lung histopathology score compared to the others in the group and the controls. This animal did not generate an antibody response to the vaccine, as the antibody levels pre-challenge were comparable to those in the unvaccinated cohort (128). Therefore, it may be that the pathology associated with this animal is linked to a lack of protection rather than VAED, which would also explain the lack of eosinophilic infiltrates usually associated with VAED. However, this case warrants further investigation.

## Discussion and Conclusions

A variety of techniques can be deployed to investigate VAED and elucidate potential mechanisms of VAED. These include traditional histopathological staining, such as H+E staining, as well as immunohistochemistry (IHC), *in situ* hybridisation (e.g. RNAscope), qPCR as well as spatial and systemic immunological analyses. There are several potential biomarkers for VAED, although the relevance of these biomarkers will vary widely depending on disease kinetics, whether the pathogen can productively infect monocytes and what immune responses are protective vs pathogenic in a particular case. These potential biomarkers following challenge could include eosinophil infiltration, weak antibody neutralisation, inflammatory monocyte infiltration, Th2-associated cytokines and immune complexes. While some of these biomarkers can be measured *in vitro*, *in vivo* evidence is required in order to demonstrate VAED. While there is no specific immunological assay for AED, assays can be developed to investigate ADE. The importance of background protection by antibodies means that an assay that measures the ratio of neutralising antibodies to enhancing antibodies, such as that developed for dengue virus, is particularly useful (23). However, observing enhanced uptake and spread of virus *in vitro* largely excludes the contributions of Fc-mediated immune functions that could contribute to elimination of infection *in vivo*, or could exacerbate pathology through induction of excessive inflammation (9). As described by Bournazos et al, concerns of ADE in Human Immunodeficiency Virus (HIV) and other viruses such as influenza and Ebola virus stemmed from *in vitro* studies but even non-neutralising antibodies with strong FcγR interactions have been shown not to mediate pathology *in vivo* and have instead been shown to be protective (9).

The use of animal models and the understanding of what aspects of these are similar or differ to humans is essential for the study of VAED (14, 15). As histopathological analysis is essential for evaluating VAED concerns, it is important that pathology is quantified so that it can be objectively assessed and compared between studies. The use of a histopathological scoring system, such as one we have described previously (16), could help to minimise overinterpretation of pathological findings and lead to the creation of standard thresholds for pathology as it relates to enhanced disease. As we have demonstrated, the timing of the post-challenge cull can influence the pathology observed (19). Including groups for short-term pathology vs long-term pathology could be useful for determining how severe clinical consequences are likely to be at particular timepoints if the pathology was replicated in humans. In order to do this, it is important to dissect the importance of transient pathology that is later resolved so that decisions can be made based on the acceptable level of transient pathology if long-term protection against severe disease is achieved. In many cases of VAED observed at a particular timepoint, protection against severe disease has also been noted. It will also be useful to compare vaccinated post-challenge models of pathology with challenge and re-challenge models. The lack of re-challenge studies in SARS-CoV-1 and MERS-CoV makes it more difficult to determine the mechanism and consequences of potential VAED. For instance, if similar pathology is seen in both possible VAED and in re-infection models, and this pathology is greater than that seen in unvaccinated animals, the immune system is likely to be enhancing infection. However, if vaccinated animals have transient pathology upon challenge, but re-infected animals have more severe pathology that is long-lasting, the vaccine is having a protective effect and preventing disease enhancement seen in natural reinfection. With some viruses, such as dengue virus, pathology is often more pronounced during re-infection than during initial infection (37), so reinfection models would help to answer the question of whether or not vaccines that enhance disease do so in a similar way to the natural course of the immune response. If not, then a component of the vaccine preparation may be to blame.

T cell contributions to VAED are generally thought to occur through Th2 cells mediating inflammation and pulmonary eosinophil infiltration. Th1 responses to vaccinations are preferable for dealing with viral infections without inducing pathology. In mouse studies, it is known that C57BL/6 mice are predisposed towards Th1-biased immune responses, while BALB/c mice are predisposed towards Th2-biased responses. Therefore, in cases of possible VAED in murine models, authors should highlight how their choice of model may have impacted any observed pathology.

For SARS-CoV-2 and other pathogenic coronaviruses, there is no vaccine preparation that is used as a control for inducing VAED. We previously attempted to create such a control for SARS-CoV-2 with a formalin-inactivated vaccine that included alhydrogel, with the goal of inducing non-neutralising antibodies and skewing T cells towards Th2 phenotypes (19). Others have also attempted to induce VAED by administering a formalin-inactivated vaccine shortly before or after challenge, when levels of circulating antibodies are likely to be quite low (20). Other ways to create a model of VAED may be to use monoclonal or polyclonal recombinant non-neutralising antibodies, with species-matched Fc regions, which could give insights into ADE and AED specifically. Recombinant antibodies against particular ‘enhancing epitopes’ such as those potentially found in SARS-CoV-1 (86) could also be used to create such a model. Ideally, a model of VAED should also take differences between homologous and heterologous challenge into account. For some viruses, such as dengue virus, differences between serotypes mean that antibodies that were neutralising against one serotype can be non-neutralising against another, mediating enhanced disease. In the context of SARS-CoV-2, this could be evaluated for different circulating variants, although the differences between virus variants are more subtle than those between virus serotypes. For *in vitro* assays examining antibody binding to viral antigens, it is important to keep the viral antigen in its native state, particularly as specific epitopes elicit neutralising antibodies whereas others may be able to confer disease enhancement. Direct coating of antigens onto ELISA plates for example, can alter the structure of antigens, as can formalin inactivation (19, 129). In contrast, capture ELISA platforms can ensure that an antigen is not modified and so responses to all epitopes can be assessed.

Earlier in the SARS-CoV-2 pandemic, another fear around VAED concerned cross-reactivity between other coronaviruses and SARS-CoV-2. One hypothesis suggested that COVID-19 may be more severe in adults than in children because adults had been exposed to a wider array of coronaviruses throughout their lifetime and as a result were producing cross-reactive but non-neutralising antibodies against SARS-CoV-2, which could cause ADE (130). Similarly, the authors also suggested imprinting as a potential reason as to why IgG responses appeared to develop much faster in many patients than they do during the course of other viral infections (130). Imprinting (also known as original antigenic sin or the Hoskin’s effect) occurs on first exposure to an immunodominant antigen on a virus, through infection or vaccination, and prevents the development of robust immune responses to other antigenic determinants on the same virus or its variants upon subsequent exposures/vaccinations. This phenomenon has been very well-characterised in antibody responses against influenza virus in particular, where it has been shown to be both protective and pathogenic, depending on how distantly related an encountered virus is from that which has been ‘imprinted’ (131). Several hypotheses have been proposed to explain its mechanism (131). Imprinting in T cell responses is however a much more controversial area. In dengue virus, it is unknown whether cross-reactive T cells are more protective or pathogenic and this may depend on the HLA alleles of individuals (132). Imprinting and cross-reactive pathogenic T cell responses were first proposed for lymphocytic choriomeningitis virus (133). The idea of imprinting and T cells has come under scrutiny, as some argue against its existence from a mechanistic standpoint, stating that T cell receptors (TCRs) with poor affinity for a variant will not be able to outcompete TCRs with a higher affinity (134).

SARS-CoV-2 vaccines have not been associated with significant VAED in preclinical studies or clinical use. It is unknown why this is the case, considering VAED was associated with other types of human coronavirus in preclinical studies. We suggest that some of the reasons we have highlighted, from formaldehyde fixation & cellular debris contaminants in formulations of experimental vaccines to over-interpretation of pathology, may explain much of the VAED in the literature. It may be that vaccines against SARS-CoV-2, even in preclinical studies, were better at avoiding VAED because of improvements in vaccine preparation. Experiments that demonstrated VAED for SARS-CoV-1 preclinical vaccines should be repeated so that these concerns can be re-evaluated.

There is potential for future variants/serotypes of viruses, including SARS-CoV-2, to provoke sub-neutralising antibodies in individuals who have encountered similar (but poorly cross-reactive) epitopes. This was the case for the SARS-CoV-2 variant known as Omicron, which demonstrates a 41-fold drop in neutralising antibody titres in patients who have received two doses of the mRNA vaccine BNT162b2 (135). Despite this drop in neutralisation, no enhancement of disease has been reported. Infection with other variants of SARS-CoV-2 have also been shown to impact antibody binding to SARS-CoV-2 and its variants post-vaccination through imprinting, but no disease enhancement has been reported in these cases either (136). Seasonal coronaviruses also appear to provide a level of back-boosting or cross-protection in some individuals (137, 138). Cross-reactivity has been observed between SARS-CoV-1 and SARS-CoV-2, which results in improved vaccine-induced immune responses by provoking the generation of broadly-neutralising antibodies against a wide variety of coronaviruses (139). However, imprinting may also have negative consequences for future vaccines based on spike proteins from SARS-CoV-2 variants, due to back-boosting of conserved but non-neutralising epitopes (140). Waning antibody levels, which are a cause of ADE in dengue virus infection, have been observed 6 months following vaccination (141, 142), however a level of protection is still being observed to date in vaccinated people and there have been no documented cases of ADE owing to this or any other cause in SARS-CoV-2. Memory T cells induced in response to vaccination have been shown to have highly heterogenous antigen-specific responses, which are thought to contribute to long-term protection against severe disease (143). Even with robust antibody escape as seen in Omicron, T cell responses are likely to be sustained (144).

Overall, genetic vaccine platforms (mRNA and viral vectors) are in theory be less likely to induce AED than inactivated vaccines or natural infection. This is because genetic platforms ensure responses are generated against unmodified neutralising epitopes, encoded by the platform, while inactivated whole-virus vaccines have a wider variety of epitopes for the immune system to generate responses against. Some of these epitopes will be non-neutralising and could therefore contribute to AED. Formalin-inactivated vaccines also pose the risk of altering the structure of antigens, as we have shown for the SARS-CoV-2 spike, however we demonstrated that this structural change does not lead to VAED in NHPs (19). The concern of formalin inactivation stimulating Th2 pathology through carbonyl group formation, which was demonstrated in mouse models of RSV (34), was also not observed in our study. Genetic vaccine platforms also have the advantage of using host-generated glycosylation when the protein of interest is synthesised, which mimics the case in natural infection. Protein-based vaccines which use non-human cell lines to produce the protein of interest could generate viral proteins with glycosylation patterns that differ from that produced during natural infection. Grant et al. found that glycans shield approximately 40% of the SARS-CoV-2 spike protein surface, with implications for human leucocyte antigen (HLA) complex binding and antigen-specific immune responses as a result (145). Many influenza vaccine antigens are produced in fertilised chicken eggs, and a glycosylation site in H3N2 influenza has been found to alter antibody binding such that weak neutralising responses to this site were elicited by both ferrets and humans (64). As differential glycosylation patterns between vaccine antigens and wild-type antigens can induce weakly neutralising antibodies in response to particular epitopes, this is another theoretical concern for VAED, provided the pathogen in question can facilitate ADE or AED.

VAED is a concern that should be carefully evaluated for new vaccines. While the term VAED is usually associated with viral infections, the phenomenon also has potential to occur with other microbial infections, which show potential for ADE and/or AED (12, 146). While VAED is a rare phenomenon, it should be studied in greater detail in preclinical models considering its clinical consequences, its potential to stall vaccine development and its ability to undermine public confidence in safe & effective licenced vaccines. We have outlined the potential mechanisms of VAED and described improved methods that can be employed so that potential problems can be identified at a pre-clinical stage and potentially false VAED signals, resulting in delays in vaccine development, can be avoided.

## Author Contributions

CG wrote the manuscript and designed the tables and figures. TT, FJS, QS, AG, and MC provided guidance and revised the manuscript. FJS, contributed to the discussion of histopathology. All authors contributed to the article and approved the submitted version.

## Funding

TT and MC were funded by the US Food and Drug Administration 75F40120C00085: Characterization of severe coronavirus infection in humans and model systems for MCM development and evaluation. TT and MC were also funded by a grant from the Coalition for Epidemic Preparedness Innovations (CEPI): Applying systems immunology to NHP COVID-19 vaccine challenge study data sets to define Correlates of Protection to SARS-CoV-2.

## Conflict of Interest

The authors declare that the research was conducted in the absence of any commercial or financial relationships that could be construed as a potential conflict of interest.

## Publisher’s Note

All claims expressed in this article are solely those of the authors and do not necessarily represent those of their affiliated organizations, or those of the publisher, the editors and the reviewers. Any product that may be evaluated in this article, or claim that may be made by its manufacturer, is not guaranteed or endorsed by the publisher.
